# Effects of Oral Cannabidiol on Health and Fitness in Healthy Adults: An 8-Week Randomized Trial

**DOI:** 10.3390/nu15122664

**Published:** 2023-06-07

**Authors:** Victoria A. Flores, Jacob N. Kisiolek, Arjun Ramani, Ryland Townsend, Edwin Rodriguez, Blake Butler, Laura K. Stewart

**Affiliations:** 1 Department of Kinesiology, Nutrition, and Dietetics, University of Northern Colorado, Greeley, CO 80639, USA; jacob.kisiolek@path.utah.edu (J.N.K.); arjun.ramani@unco.edu (A.R.); ryland.townsend@unco.edu (R.T.); edwin.rodriguez@unco.edu (E.R.); butler.blake14@gmail.com (B.B.); 2 Department of Kinesiology and Nutrition, University of Illinois at Chicago, Chicago, IL 60612, USA; 3 Department of Pathology, Division of Microbiology and Immunology, University of Utah, Salt Lake City, UT 84112, USA

**Keywords:** cannabinoids, healthy adults, physical performance, cognition, psychological wellbeing

## Abstract

Background: There is a lack of research on the effects of cannabidiol (CBD) on health-related fitness, physical activity, cognitive health, psychological wellbeing, and concentrations of C-reactive protein (CRP) in healthy individuals. CBD has potential anti-inflammatory and neuroprotective effects. Methods: This study aimed to investigate the effects of 8 weeks of CBD on the above-mentioned measures in healthy individuals. Forty-eight participants were randomized into two groups receiving either oral capsules of 50 mg of CBD or a calorie-matched placebo daily. Participants completed pre- and post-intervention assessments, including blood draws, body composition, fitness, physical activity, and self-reported surveys. Results: There were no significant differences between groups regarding body composition, aerobic fitness, muscular strength, physical activity, cognitive health, psychological wellbeing, and resting CRP concentrations. However, the placebo group experienced a decline in mean peak power and relative peak power compared to the CBD group. Conclusions: The results suggest that 8 weeks of CBD supplementation may prevent declines in anaerobic fitness over time. However, long-term CBD supplementation may not be beneficial for altering measures of health-related fitness, mental health, and inflammation in healthy individuals.

## 1. Introduction

Cannabidiol (CBD), the non-intoxicating phytocannabinoid contained in *Cannabis sativa* L. has significant therapeutic potential for the treatment of neuroinflammatory diseases and disorders including depression [1,2,3,4,5], anxiety [1,3,4,5], Parkinson’s disease [6], Alzheimer’s disease [5,6], and a variety of cancers [3]. CBD is proposed to exert these effects through various mechanisms involving the central nervous system, including interacting with cannabinoid receptors (CB1 and CB2) [7], modulating neurotransmitter systems, such as serotonin (5-HT) and gamma-aminobutyric acid (GABA) [8], influencing ion channels (TRPV1, TRPV2) [9], acting as an allosteric modulator of GABA receptors, and activating nuclear receptors (PPARγ) [10], which in turn, may impact cognitive function, neuronal plasticity, and neurotransmitter balance [8]. Currently, there is one Food and Drug Association (FDA)-approved CBD product, Epidiolex, prescribed at 2.5 to 20 mg/kg/day to treat infants and adults with treatment-resistant seizures [11], and recent research suggests that CBD has the potential to control adult complex onset seizures as an adjunct therapy by reducing seizure burden [12]. CBD has not been widely tested for consumers without neuroinflammatory diseases and disorders and is marketed for use in a wide variety of health- and performance-related reasons. For instance, consumers online positively view CBD products in terms of therapeutic effects and as a topical cosmetic [13]. Elite rugby players report taking 400–3000 mg of CBD for exercise recovery and improved sports performance [14]. Additionally, adults aged 18–34 years old report taking less than 50 mg daily for self-perceived general wellbeing and post-workout soreness [15].

CBD manufacturers encourage daily CBD use for physical health and fitness, despite mixed evidence for the support of CBD in measures of health-related fitness and physical activity. Randomized controlled trials (RCTs) investigating acute CBD ingestion following a muscle damage protocol concluded that oral CBD in dosages of 2–10 mg/kg, 60 mg, and 150 mg has no effect on recovery, torque, and strength and power performance [16,17,18,19]. It is unknown whether long-term CBD use influences anaerobic performance without a muscle damaging protocol or alters other aspects of health and fitness. Some studies suggest that CBD may play a role in body composition through glucose metabolization in adipocytes [20] and brown adipose tissue [21], but one study suggests that oral CBD (30 mg) has no effect on body composition in those with overweight or obesity [22]. One RCT observed that acute CBD ingestion (300 mg) increased maximal oxygen consumption (VO_2_ max) without altering other cardiovascular parameters [23], but there are no other studies to confirm this aerobic enhancement effect. Regarding improvements in physical activity, only one canine study investigated the effects of treats made with 25 mg of CBD on physical activity behavior for 7 days and observed no changes in voluntary walking and running [24].

The impact of daily CBD consumption on mental health and inflammation in healthy individuals remains understudied. In patients with cognitive deficits, 6 weeks of 1000 mg/day of oral CBD improved memory and learning [25], while a single dose of 600 mg of oral CBD increased cerebral blood flow, but did not affect memory and learning in healthy adults without cognitive impairment [26]. No research has investigated the effect of long-term CBD consumption at doses lower than clinical recommendations (e.g., 30 to 1400 mg/day [11]) in healthy adults, and whether consumption alters cognitive function and psychological wellbeing. Mental health is essential as 37% of consumers reported CBD use for general health and wellbeing [15], and 62% reported consumption for self-perceived anxiety and depression [27]. The effect of CBD on inflammatory biomarkers in physically active individuals is inconclusive. One RCT observed that 12 weeks of 400–800 mg/day of CBD reduced concentrations of IL-6 in adults diagnosed with cocaine use disorder [28]. Additionally, another study observed that 3 weeks of 67 mg/day of CBD oil was associated with decreased expression of proinflammatory genes and reductions in *C*-reactive protein (CRP) in human immunodeficiency virus (HIV)-positive participants [29]. Conversely, another RCT observed no effect on IL-6 and CRP after 13 weeks of 200 mg/day of CBD in adults with type 2 diabetes [30].

The lack of knowledge surrounding the effects of CBD on physical fitness, mental health, and inflammation is concerning for physically active individuals seeking to use CBD for unsupported health claims. This study aimed to investigate the effects of an 8-week oral CBD intervention on health-related fitness, physical activity patterns, cognitive health, psychological wellbeing, and resting CRP concentrations in physically active adults. We hypothesized that CBD consumption would lead to improvements in aerobic and anaerobic fitness, increased average daily step counts, improved cognitive function and psychological wellbeing scores, and lower resting concentrations of CRP compared to the placebo group. This hypothesis stems from previous studies suggesting the potential of CBD to modulate inflammatory responses, mental health, and overall wellbeing, as well as its potential effects on physical performance and activity levels in preclinical and limited human studies.

## 2. Materials and Methods

### 2.1. Study Overview

This double-blind, randomized, placebo-controlled trial was registered with ClinicalTrials.gov (Registration: NCT04881539). Participants completed eight visits: four pre- and four post-intervention visits, with an 8-week intervention period in between. During the first pre-intervention visit, participants reviewed and signed the written informed consent form. Participants were given an activity tracker (Fitbit, San Francisco, CA, USA) to wear for seven days before and during week 7 for one final week of the intervention. Participants then completed an 8-h fasted blood draw, cognitive function and psychological wellbeing scales, and a body size and composition assessment. Pre-intervention visits 2–4 were conducted on separate days. Participants completed an aerobic fitness test assessing relative peak oxygen uptake (VO_2_ peak) on visit 2, an anaerobic fitness test assessing anaerobic power output on visit 3, and a muscular strength test assessing back squat and bench press one-repetition maximums (1RMs) on visit 4. During the 8-week intervention, participants consumed either 50 mg of hemp-derived CBD (Six Degrees Wellness, Boulder, CO, USA; Supplementary Material) or 225 mg of medium-chain triglyceride (MCT; Nutiva, Point Richmond, CA, USA) as a calorie-matched placebo daily, after dinner and before bed. Adverse events were assessed throughout the study period using participant self-reporting and regular check-ins with the research team to monitor any potential adverse effects associated with the CBD or placebo administration. During each visit, participants were encouraged to communicate any discomfort, side effects, or changes in their health status to the research team for proper evaluation and documentation. Participants were instructed to abstain from all other cannabis products and not to discuss the study nor its supplements with other individuals. Participants completed the same pre-intervention assessments in post-intervention visits 5–8. Primary outcomes included body mass (BM), lean body mass (LBM), body fat percent (BF%), relative VO_2_ peak, peak and relative peak power (PP and RPP), mean and relative mean power (MP and RMP), anaerobic fatigue (AF), back squat and bench press 1RM, 7-day average of steps/day, self-reported cognitive function and psychological wellbeing scores, and resting concentrations of CRP (mg/L).

### 2.2. Subjects

All subjects gave their informed consent for inclusion before they participated. The study was conducted in accordance with the Declaration of Helsinki, and the protocol was approved by the Ethics Committee of the University of Northern Colorado Institutional Review Board (IRB). All participants met the following inclusion criteria: 18–50 years old, 6 weeks of abstinence from cannabis (either tetrahydrocannabinol (THC) and/or CBD), and no chronic alcohol and/or drug use. Participants were excluded if they were diagnosed with cardiovascular, neurological, metabolic, or mood disorders, were pregnant and/or nursing, or were unable to adhere to an 8-week supplement intervention. All participants were informed of the benefits and risks of the investigation prior to signing the IRB-approved consent form. Participants were randomly assigned following simple randomization procedures to 1 of 2 treatments: CBD intervention group (CG) or placebo group (PG). Randomization and supplement allocation was conducted by an independent investigator with no involvement in the recruitment, consent, or data collection and analysis processes.

### 2.3. Procedures

#### 2.3.1. Body Size and Composition Assessments

Height was measured using a stadiometer (SECA 220, Chino, CA, USA) and BM was measured using a digital scale (Detecto, Webb City, MO, USA). LBM and BF% were measured with a calibrated BodPod (Cosmed Inc., Concord, CA, USA).

#### 2.3.2. Aerobic Fitness Assessment

A refractometer (Atago, Tokyo, Japan) was used to evaluate urine specific gravity prior to aerobic fitness testing with a dehydration cutoff point set to ≥1.020 mg/dL. Dehydrated participants were asked to hydrate and reschedule if dehydration persisted. A treadmill (Trackmaster, Full Vision Inc., Newton, KS, USA), customized protocol, and metabolic cart (Parvomedics TrueOne, Sandy, UT, USA) were used to evaluate relative VO_2_ peak. The customized protocol was based on the modified Åstrand test and complied with directives provided by the American College of Sports Medicine Guidelines (ACSM) for multistage exercise testing [31]. The test began with a 5-min warm up, followed by 3-min stages of progressive, graded exercise, and ended with a cool-down once participants reached volitional fatigue.

#### 2.3.3. Anaerobic Fitness Assessment

Anaerobic power was evaluated using the 30-s Wingate test [32] on a cycle ergometer (Monark, Varberg, Sweden). The test began with a 5-min warm-up, followed by the 30-s test with 7.5% of their body weight, and ended with a mandatory cool-down of at least 5–10 min.

#### 2.3.4. Muscular Strength Assessment

Upper and lower body muscular strength was assessed with guidance from the National Strength and Conditioning Association (NSCA) [33]. The test began with a warm-up (1 set of 8–10 reps with an unloaded barbell), followed by a warm-up consisting of 30–60% of the body weight added to the bar (1 set of 3–5 reps), and a working set of 80–95% of body weight added to the bar (1 set of 1–2 reps). Participants rested for at least 2–4 min between 1RM attempts. Weight was only added to achieve 100% of each participant’s 1RM. If the participant successfully completed the lift at this weight, additional weight was added until the participant failed to lift the weight.

#### 2.3.5. Physical Activity Assessment

Participants were given a Fitbit to wear with notifications turned off, and black tape covering the device surface to discourage changes in physical activity behavior. Participants were instructed to proceed with their normal physical activity and to wear the tracker at all hours during the pre-intervention week and week 7 of the 8-week intervention.

#### 2.3.6. Measures of Mental Health and Wellbeing

Subjective cognitive function was evaluated with the National Institute of Health Patient-Reported Outcomes Measurement Information System (NIH PROMIS) Cognitive Function–Abilities—Short Form 8a and objective function was measured with the NIH PROMIS Cognitive Function—Short Form 8a [34]. Each item assessed participant-perceived facets of cognitive function and abilities, including mental acuity, concentration, verbal and nonverbal memory, verbal fluency, and perceived changes in these cognitive functions [34]. Raw scores from both short forms were reported as T-scores using the NIH PROMIS grading tool. Subjective wellbeing was measured with the psychological wellbeing scale that assessed 6 aspects of wellbeing including autonomy, environmental mastery, personal growth, positive relation with others, purpose in life, and self-acceptance with a 7-point Likert scale [35]. Scores were determined by summing all items within each subscale with higher scores indicating greater wellbeing.

#### 2.3.7. Blood Collection and CRP

Participants were instructed to avoid strenuous physical exercise for 48 hours before each blood draw and completed a 24-h diet recall prior to blood collection in visit 1 in which they were asked to repeat prior to blood collection in visit 5. Whole blood was collected into serum separator tubes (Beckton Dickinson, East Rutherford, NJ, USA) by a certified phlebotomist, allowed to clot for 30-min at room temperature, then centrifuged at 2000 RPM for 15 min. The serum was pipetted into 1.5 mL microcentrifuge tubes (Eppendorf AG, Hamburg, Germany) and immediately stored in a −80 °C freezer. Serum concentrations of CRP were determined with a commercially available enzyme-linked immunosorbent assay (ALPCO Diagnostics, Salem, NH, USA). Microplates were read with an ELx800 BioTek microplate reader (BioTek Instruments, Inc., Winooski, VT, USA) at the recommended wavelength of 450 nanometers.

### 2.4. Statistical Analyses

To achieve a desired level of 0.80 power with an *α* = 0.05, an a priori analysis (G*Power, Dusseldorf, Germany) using means and standard deviations (SDs) of pre- and post-intervention concentrations of CRP in physically active adults were used for computational analyses [36]. A total sample size of 36 was needed, but was increased to at least 48 to anticipate a ~25% dropout rate. A mixed analysis of variance (ANOVA) was used to determine the interaction effects of time (within-subjects) and treatment group (between-subjects) on the primary outcomes. The data were evaluated for outliers by boxplot inspection and removed if outliers were ±2.5 SD from the mean. Normality was assessed using the Shapiro–Wilk’s test (*p* > 0.05), homogeneity using the Levene’s test for equality of variances (*p* > 0.05), and sphericity using the Mauchly’s test of sphericity (*p* > 0.05). Pairwise comparisons were performed with the Bonferroni post hoc test where significant interactions were detected. Effect sizes for the F-statistic were expressed as partial eta squared (*η^2^*) and values of 0.01, 0.06, and 0.14 were interpreted as small, medium, and large effects, respectively [37]. An independent-samples t-test was used to compare means of participant characteristics with a 95% confidence interval and significance set to *p* < 0.05. All statistical analyses were performed with SPSS 25 (IBM, Corp., Chicago, IL, USA).

## 3. Results

### 3.1. Subjects

**Figure 1:**
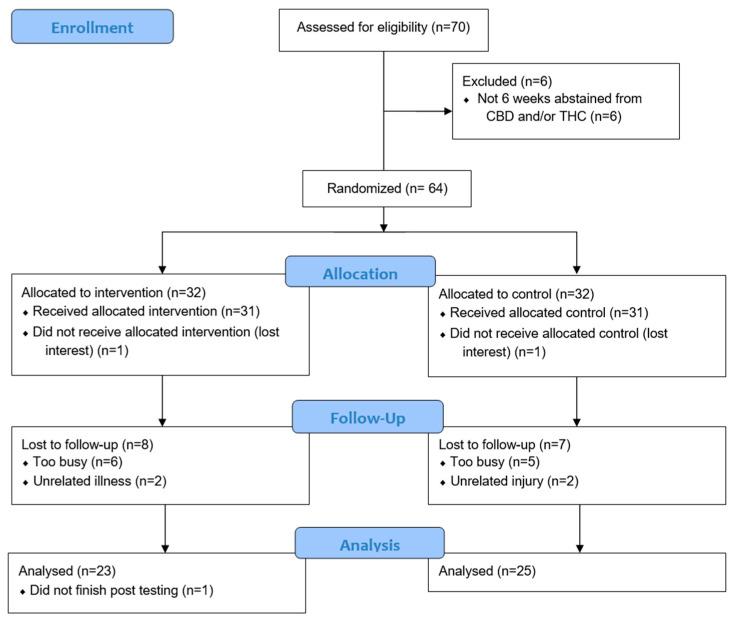
Consort Flow Diagram. CBD = cannabidiol, THC = tetrahydrocannabinol.

Forty-eight participants (CG: *n* = 23; PG: *n* = 25; males: *n* = 24; females: *n* = 24; males in CG: *n* = 12; males in PG: *n* = 12; females in CG: *n* = 11; females in PG: *n* = 13) completed the study (Figure 1). Participants’ means and SDs for age, height, and BM, were 25 ± 6 years, 171 ± 10 cm, and 73 ± 13 kg, respectively. There were no severe adverse events or reports of supplement intolerance, and no differences between treatment groups with respect to participant characteristics and resting cardiovascular measures (Table 1). When both groups were combed, age ranged from 18 to 42 years, height ranged from 152 to 195 cm, and BM ranged from 19.6 to 33 kg.

### 3.2. Health-Related Fitness

There were no treatment differences with respect to body size and composition, aerobic fitness, and muscular strength measures over the course of the intervention (Table 2). When both groups were combined, LBM ranged from 36.8 to 83.5 kg, and BF% ranged from 8.2 to 39.7%, relative VO_2_ peak ranged from 27.7 to 62.3 mL/kg/min, bench press 1RM ranged from 25 to 161 kg, and back squat 1RM ranged from 39 to 166 kg.

**Figure 2:**
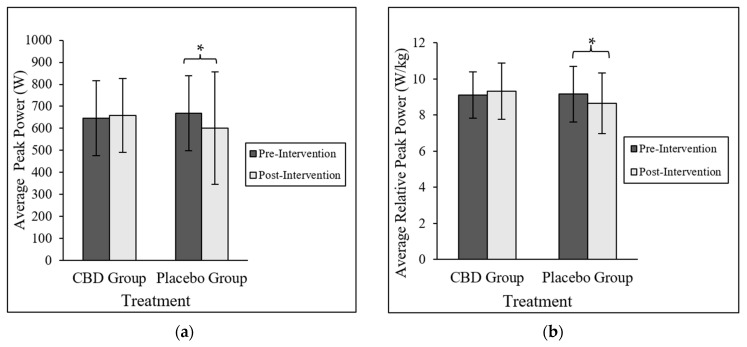
Anaerobic Power. Data are presented as mean ± SD. (**a**) Pre- and post-intervention mean peak power. Data are presented as mean ± SD. * Indicates that PG peak power post-intervention was significantly lower than PG peak power pre-intervention compared to CG peak power pre- and post-intervention (Bonferroni adjusted *p* = 0.006). (**b**) Pre- and post-intervention mean relative peak power. Data are presented as mean ± SD. * Indicates that PG relative peak power post-intervention was significantly lower than PG relative peak power pre-intervention compared to CG relative peak power pre- and post-intervention (Bonferroni adjusted *p* = 0.006).

A significant interaction was found with respect to PP (*p* = 0.045; *η^2^* = 0.110; Figure 2a) and RPP (*p* = 0.025; *η^2^* = 0.136; Figure 2b). A Bonferroni post hoc analysis confirmed that PG experienced a 9.6% decrease in PP (*p* = 0.006) and a 6.6% decrease in RPP (*p* = 0.006) compared to CG, whereas CG experienced no changes in either PP and RPP post-intervention. There were no treatment differences with respect to MP, RMP, and AF (Table 3). When both groups were combined, PP ranged from 372.1 to 1147.7 W, RPP ranged from 6 to 12.6 W/kg, MP ranged from 287.8 to 803.9 W, RMP ranged from 4.2 to 8.3 W/kg, and AF ranged from 43.3 to 71.3%.

### 3.3. Physical Activity Measures

The mean ± SD pre-intervention steps/day were 11,846 ± 4119 steps/day for CG and 11,019 ± 4286 steps/day for PG, and the mean ± SD post-intervention steps/day were 11,125 ± 3362 steps/day for CG and 10,787 ± 4000 steps/day for PG. There were no differences between treatment groups. When both groups were combined, the 7-day average was 11,415 ± 4183 steps/day and ranged from 6535 to 24,971 steps/day.

### 3.4. Measures of Mental Health and Wellbeing

Self-reported cognitive function and psychological wellbeing are displayed in Table 4 and Table 5, respectively. There were no treatment differences between groups. When both groups were combined, cognitive function T-scores ranged from 29.8 to 63.9 and cognitive function ability T-scores ranged from 36.9 to 67.1. Regarding the aspects of psychological wellbeing, autonomy scores ranged from 8 to 21, environmental mastery scores ranged from 7 to 21, personal growth scores ranged from 15 to 21, scores for positive relation with others ranged from 10 to 21, purpose in life scores ranged from 11 to 21, and self-acceptance scores ranged from 8 to 21.

### 3.5. Resting Concentrations of CRP

The mean ± SD concentrations of CRP pre-intervention were 1.5 ± 2 mg/L for CG and 1.3 ± 1.6 mg/L for PG, and the mean ± SD concentrations of CRP post-intervention were 1.3 ± 1.6 mg/L for CG and 1.6 ± 2 mg/L for PG. There were no treatment differences with respect to resting concentrations of CRP. When both groups were combined, the mean ± SD concentration of CRP was 1.4 ± 1.8 mg/L and ranged from 0.1 to 8.8 mg/L.

## 4. Discussion

Contrary to our hypothesis, 8 weeks of CBD supplementation did not lead to improvements in aerobic and anaerobic fitness, physical activity, mental health and wellbeing, and inflammation. However, the present study revealed a potential effect of CBD on power output. CBD appeared to prevent reductions in peak anaerobic output in physically active adults, as evidenced by PG experiencing a significant ~10% decrease in PP, while CG experienced a non-significant ~3% increase in PP at the end of the intervention. This translated to a ~7% decrease in RPP for PG, but a ~3% increase in RPP for CG.

The observed outcome of CBD preventing reductions in peak anaerobic output may be explained by the combination of CBD and exercise-induced oxidative stress. In a study on mice subjected to 3 weeks of exercise training, CBD treatment (50 mg/kg) down-regulated inflammatory protein expression and reversed myocardial injury [38]. It is possible that the recreationally active participants in this study, who had favorable step counts, experienced a similar effect that aided anaerobic power. CBD is proposed to exert its antioxidant activity through both direct and indirect mechanisms [8]. The molecular structure of CBD, with its aromatic nucleus and hydroxyl group on the phenolic nucleus, contributes to its antioxidant properties by converting reactive species into less reactive compounds [39,40]. Additionally, CBD influences redox homeostasis by decreasing the production of reactive oxygen species (ROS) through chelating transition metal ions involved in oxidative reactions and increasing the gene expression of endogenous antioxidant systems, such as superoxide dismutase (SOD) and glutathione peroxidase (GPx), via the Nrf2/Keap1 complex [41,42,43]. However, the proposition that improved antioxidant levels resulted in a protective effect on anaerobic power is speculative since resting oxidative stress markers were not evaluated, and no studies exist on long-term CBD consumption and anaerobic fitness performance.

The results of the present study suggest that CBD may not influence measures of body composition. It was hypothesized that CBD would affect body composition by reducing BF%, based on research suggesting that CBD reduces intramuscular fatty acid accumulation, inhibits de novo lipogenesis, and improves fatty acid metabolism in both oxidative and glycolytic muscle types in rat models of obesity [44]. However, the findings from the present study are consistent with previous human research. One study, which included overweight and obese males assigned to either 6 weeks of 15 mg of daily CBD in a hemp oil extract or a placebo, found no differences in LBM [45]. Additionally, another study including a similar population of males with overweight and obesity found no changes in metabolic function after acute 30 mg CBD ingestion in a variety of CBD formulations [22]. It is possible that longer observation times and with higher doses of CBD are necessary to induce measurable changes in body composition. It is also possible that the anti-inflammatory effects of CBD which potentially modulate metabolic regulators in muscle are only observable in preclinical studies [46]. The effect of long-term CBD consumption on LBM and BF% in humans is understudied and is only addressed in survey-based cannabis research. For example, in a survey study of 50,000 adult cannabis users, high-frequency cannabis users had 14–17% lower obesity prevalence compared to 22–25% in non-users [47]. This finding suggests that this disparity in body composition may be due to cannabinoids other than CBD.

The influence of CBD on aerobic and muscular strength measures was investigated, and no significant differences were observed. While a previous RCT observed that acute CBD (300 mg) intake increased VO_2_ max (+0.1 ± 0.2 L/min) without increasing heart rate, rate of perceived exertion, or time to exhaustion in endurance-trained men [23], relative VO_2_ peak was not affected in the present study. The aforementioned study also observed reduced concentrations of anandamide immediately post-test, suggesting a possible mechanism of action for CBD to confer cardiorespiratory benefits through the endocannabinoid system [23]. Preclinical models suggest that extreme acute stress, whether drug- or exercise-induced, must be elicited for CBD to exert mitigating effects [38]. It is possible that the VO_2_ peak assessment in the present study was not strenuous enough to observe an effect of CBD on aerobic capacity. Additionally, no effect of CBD was observed on muscular strength measures, which is similar to muscle damaging protocol studies that observed no effect of CBD on torque, strength, and power performance [16,17,18,19]. The present study did not subject participants to exercise-induced muscle damage and tested muscular strength with an NSCA-guided protocol, which allowed recovery periods between maximal attempts. It is unclear whether CBD plays a role in enhancing muscular strength and performance or if it acts through other signaling pathways beyond skeletal muscle function.

Cognitive function, psychological wellbeing, and inflammation did not improve in CG compared to PG, and this observation was contrary to previous research [25]. When the sample size was compared to other healthy adults, mean cognitive function scores were categorized as “typical”, with males 3% below the male mean, and females 1% above the female mean [48]. There were no differences with respect to the six aspects of psychological wellbeing. However, a main effect of time revealed that mean scores for personal growth, positive relation with others, and purpose in life significantly decreased by 5%, 7%, and 7% by the end of the intervention (*p* < 0.001, *p* = 0.017, and *p* = 0.016, respectively), possibly due to the timing of data collection during the pandemic. Resting concentrations of CRP did not improve, regardless of individual pre-intervention cardiovascular disease (CVD) risk stratification. According to CVD risk stratification literature [49], 50% of participants in the present study were in the low-risk category (serum CRP < 1 mg/L), 24% of participants were in the moderate-risk category (serum CRP 1–3 mg/L), and 15% were in the high-risk category for CVD (serum CRP > 3 mg/L). It is possible that other dosages or cannabinoid mixtures are required for CBD to influence mental health and inflammation.

There are several limitations that must be considered. The absence of specific measurement and randomization regarding the participants’ level of education and number of hours of physical activity per week before the study may have influenced the response to study procedures and assessments. Additionally, the surveys used to measure cognitive function may not have fully captured the diverse range of cognitive functions and abilities. Including more specific scales targeting attention, working memory, and executive functions would have provided a more comprehensive evaluation of cognitive performance. Another limitation of our study is that the 8-week intervention may not have been sufficient to observe significant effects on physical and mental health in healthy adults. The 8-week intervention duration was based on clinical trials involving oral CBD [25,50]. It is important to note that CBD may have differential effects on physical and mental health outcomes in neuropsychiatric populations relative to healthy populations. The daily dose of 50 mg of CBD may have been too small to observe changes in outcomes or may have resulted in participant desensitization. The dosage was chosen to reflect products available to and used by CBD consumers. Although CBD has not demonstrated a potential for abuse and is generally well-tolerated [51], there is limited information on dosages beyond 50 mg [52]. Finally, physical activity and exercise training were not monitored nor evaluated during the intervention period. Unreported changes may have contributed to differences observed in subgroup analyses of sex, treatment, and time. Future studies may benefit from addressing the limitations of the present study, such as investigating different CBD dosages and combinations of cannabinoids to observe changes in physical and mental outcomes. Additionally, monitoring and evaluating physical activity and exercise training during the intervention period may help to account for potential unreported changes.

Despite the limitations mentioned, this study has notable strengths. First, it employed a rigorous double-blind, randomized, placebo-controlled design. Second, a comprehensive set of assessments including measures of body composition, aerobic and anaerobic fitness, cognitive function, psychological wellbeing, and inflammation were used. This multidimensional approach provides a holistic view of the potential effects of CBD on various aspects of health and fitness. Lastly, the study recruited a diverse sample of physically active adults, which increases the generalizability of the findings to a broader population. 

## 5. Conclusions

The present study observed that daily consumption of 50 mg of CBD for 8 weeks did not result in significant improvements in body composition, aerobic and other muscular strength measures, mental health, or inflammation in physically active adults. However, CBD supplementation appeared to attenuate decreases in peak anaerobic power over time. The study also observed a possible effect of CBD on average power output, which warrants further investigation. The limitations of the present study should also be considered when interpreting the results. Future studies should consider longer intervention durations, higher CBD doses, and monitoring physical activity and exercise training during the intervention period. Overall, these findings contribute to the limited knowledge surrounding the effects of CBD on physical fitness, mental health, and inflammation, and highlight the need for further research to fully understand the potential benefits and limitations of CBD consumption in healthy individuals.

## Figures and Tables

**Table 1:** Participant characteristics and resting cardiovascular measures.

Variable	CG	PG	Overall	*p*
Age (years)	24.3 ± 4.5	26.5 ± 6.5	25.5 ± 5.7	0.165
Height (cm)	170.1 ± 9.7	171.2 ± 9.8	171 ± 10	0.700
Body mass (kg)	72.4 ± 15.5	73.3 ± 10.6	73.6 ± 13.7	0.884
Resting heart rate (bpm)	68 ± 7	66.2 ± 11.1	67 ± 9	0.366
Resting systolic blood (mmHg)	117 ± 8.2	118 ± 11.4	118 ± 10	0.955
Resting diastolic blood (mmHg)	74.5 ± 7.2	74.6 ± 7.3	75.1 ± 7	0.652

Data are presented as mean ± SD. CG = CBD group. PG = placebo group. cm = centimeters. kg = kilograms. bpm = beats per minute. mmHg = millimeter mercury.

**Table 2:** Pre- and Post-Intervention Body Composition, Aerobic Fitness, and Muscular Strength Measures.

Health-Related Fitness Variable	Pre-CG	Pre-PG	Post-CG	Post-PG
Lean body mass (kg)	57 ± 12.4	56.8 ± 11.1	58.8 ± 12.5	57.8 ± 12.4
Body fat percentage (%)	20.9 ± 8.3	22.1 ± 9.1	20.9 ± 8.2	23.1 ± 9.1
Relative VO_2_ peak (mL·min^−1^·kg^−1^)	45 ± 8.2	43.0 ± 7.2	45.9 ± 8.2	42.8 ± 8
Bench press 1RM (kg)	66.1 ± 31	64.5 ± 33.7	67.4 ± 30	65.7 ± 34.8
Back squat 1RM (kg)	94.6 ± 29.1	88.7 ± 34.2	98 ± 28.6	93.3 ± 32.8

Data are presented as mean ± SD. Pre-CG = pre-intervention control group. Pre-PG = pre-intervention placebo group. Post-CG = post-intervention control group. Post-PG = post-intervention placebo group. VO_2_ peak = peak oxygen uptake. 1RM = one repetition maximum. kg = kilogram. % = percent. mL = milliliters. min = minutes.

**Table 3:** Pre- and Post-Intervention Mean Power, Relative Mean Power, and Anaerobic Fatigue.

Anaerobic Output Variable	Pre-CG	Pre-PG	Post-CG	Post-PG
Mean power (W)	485 ± 127	476.7 ± 126.6	486.3 ± 126.6	467.2 ± 140.6
Relative mean power (W/kg)	6.6 ± 1	6.4 ± 1.2	6.6 ± 1	6.3 ± 1.3
Anaerobic fatigue (%)	57.1 ± 7.9	56 ± 6	58.9 ± 6.1	58.0 ± 9.1

Data are presented as mean ± SD. Pre-CG = pre-intervention control group. Pre-PG = pre-intervention placebo group. Post-CG = post-intervention control group. Post-PG = post-intervention placebo group. W = watts. W/kg = watts per kilogram. % = percent.

**Table 4:** Pre- and Post-Intervention Cognitive Function and Abilities T-Scores.

Survey	Pre-CG	Pre-PG	Post-CG	Post-PG
Cognitive Function T-Scores	49 ± 6.6	48.4 ± 9.6	48.8 ± 6.8	47.8 ± 11.8
Cognitive Abilities T-Scores	51.9 ± 7.2	51.6 ± 8.8	52 ± 8.4	51.3 ± 12.2

Data are presented as mean ± SD. Pre-CG = pre-intervention control group. Pre-PG = pre-intervention placebo group. Post-CG = post-intervention control group. Post-PG = post-intervention placebo group.

**Table 5:** Pre- and Post-Intervention Psychological Wellbeing Aspect Scores.

Wellbeing Aspect	Pre-CG	Pre-PG	Post-CG	Post-PG
Autonomy	17.1 ± 3	16 ± 3	16.6 ± 2.7	15.7 ± 3.6
Environmental Mastery	16.1 ± 2.8	15.1 ± 3.6	15.1 ± 3.8	15.5 ± 3.6
Personal Growth	20.1 ± 1.2	20.3 ± 1	19 ± 2.2	19.4 ± 2.3
Positive Relation with Others	17.3 ± 3.1	18.1 ± 2.8	16.8 ± 3	16.3 ± 3.9
Purpose in Life	17.4 ± 2.6	17.7 ± 2.1	16.4 ± 3	16.5 ± 3.9
Self-Acceptance	17.6 ± 2.7	17.8 ± 3.6	17.13 ± 2.4	17.3 ± 3.6

Data are presented as mean ± SD. Pre-CG pre-intervention control group. Pre-PG = pre-intervention placebo group. Post-CG = post-intervention control group. Post-PG = post-intervention placebo group.

## Data Availability

The data that support the findings of this study are available from the corresponding authors upon reasonable request. There are no publicly archived datasets generated or analyzed during this study.

## Supplementary Materials

The following supporting information can be downloaded at: https://www.mdpi.com/article/10.3390/nu15122664/s1. The third-party testing certificate for the CBD product was used in the present study. The certificate provides information on the purity, potency, and quality of the CBD product. The testing was conducted by an independent laboratory and the results are included in the certificate.
