# Supplementary material for: Effects of Oral Cannabidiol on Health and Fitness in Healthy Adults: An 8-Week Randomized Trial
Source: Nutrients. 2023 Jun 7;15(12):2664. doi: 10.3390/nu15122664 (PMC10301202; doi:10.3390/nu15122664)
Supplement: Supplementary file 1 [file nutrients-15-02664-s001.zip › nutrients-2426017-supplementary.pdf]

PROVERDE LABORATORIES

Test Certificate

Certificate ID: **71387**
Received: **11/22/19**
Client Sample ID: **50 mg Capsules**
Lot Number: **19021101**
Matrix: **Capsules/Tablets - Capsule-Oil Based**

Scan QR Code for authenticity

6° Wellness
1002 Walnut St., Suite 300
Boulder, CO 80302
Attn: Jonny Lisano

Authorization:	Signature:	Date:
Elizabeth R. Wagoner, Lab Director		12/2/2019

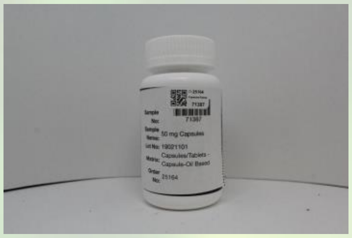

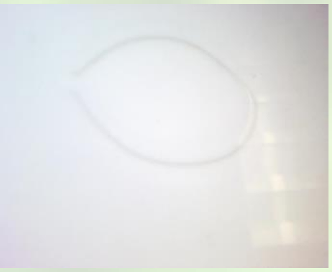

PJLA Testing
Accreditation
80585

The data contained within this report was collected in accordance with the requirements of ISO/IEC17025:2017. I attest that the information contained within the report has been reviewed for accuracy and checked against the quality control requirements for each method. These results relate only to the test article listed in this report. Reports may not be reproduced except in their entirety.

CN: Cannabinoid Profile & Potency [WI-10-17 & WI-10-17-01] *Analyst: JSG* *Test Date: 11/27/2019*

The client sample was analyzed for plant-based cannabinoids by Liquid Chromatography (LC). The collected data was compared to data collected for certified reference standards at known concentrations.

71387-CN

ID	Weight %	Concentration (mg/Capsule)
D9-THC	ND	ND
THCV	ND	ND
CBD	11.55	52.71
CBDV	0.03	0.14
CBG	ND	ND
CBC	ND	ND
CBN	ND	ND
THCA	ND	ND
CBDA	ND	ND
CBGA	ND	ND
D8-THC	ND	ND
exo-THC	ND	ND
Total	11.58	52.85
Max THC	ND	ND
Max CBD	11.55	52.71

0% Cannabinoids (wt%) 11.6%

Limit of Quantitation (LOQ) = 0.009 wt%

Max THC (and Max CBD) are calculated values for total cannabinoids after heating, assuming complete decarboxylation of the acid to the neutral form. It is calculated based on the weight loss of the acid group during decarboxylation: Max THC = (0.877 x THCA) + THC. This calculation does not include other cannabinoid isomers (eg. D8-THC and exo-THC). ND = None detected above the limits of detection (LOD), which is half of LOQ.

HM: Heavy Metal Analysis [WI-10-13] *Analyst: CJS* *Test Date: 11/25/2019*

This test method was performed in accordance with the requirements of ISO/IEC 17025. These results relate only to the test article listed in this report. Reports may not be reproduced except in their entirety.

71387-HM

Symbol	Metal	Conc. [1] (µg/kg)	RL	Use Limits [2] (µg/kg) All	Use Limits [2] (µg/kg) Ingestion	Status
As	Arsenic	ND	50	200	1500	PASS
Cd	Cadmium	ND	50	200	500	PASS
Hg	Mercury	ND	50	100	1500	PASS
Pb	Lead	ND	50	500	1000	PASS

1) ND = None detected to Lowest Limits of Detection (LLD)
2) MA Dept. of Public Health: Protocol for MMJ and MIPS, Exhibit 4(a) for all products.
3)USP exposure limits based on daily oral dosing of 1g of concentrate for a 110 lb person.

MB1: Microbiological Contaminants [WI-10-09] *Analyst: AEG* *Test Date: 11/26/2019*

This test method was performed in accordance with the requirements of ISO/IEC 17025. These results relate only to the test article listed in this report. Reports may not be reproduced except in their entirety.

71387-MB1

Symbol	Analysis	Results	Units	Limits*	Status
AC	Total Aerobic Bacterial Count	<100	CFU/g	100,000 CFU/g	PASS
CC	Total Coliform Bacterial Count	<100	CFU/g	1,000 CFU/g	PASS
EB	Total Bile Tolerant Gram Negative Count	<100	CFU/g	1,000 CFU/g	PASS
YM	Total Yeast & Mold	<100	CFU/g	10,000 CFU/g	PASS

Note: All recorded Microbiological tests are within the established limits.

MB2: Pathogenic Bacterial Contaminants [WI-10-10] *Analyst: LabAdmin* *Test Date: 11/27/2019*

This test method was performed in accordance with the requirements of ISO/IEC 17025. These results relate only to the test article listed in this report. Reports may not be reproduced except in their entirety.

71387-MB2

Test ID	Analysis	Results	Units	Limits*	Status
71387-ECPT	E. coli (O157)	Negative	NA	Non Detected	PASS
71387-SPT	Salmonella	Negative	NA	Non Detected	PASS

Note: All recorded pathogenic bacteria tests passed.

VC: Analysis of Volatile Organic Compounds [WI-10-28] *Analyst: JR* *Test Date: 11/22/2019*

The client sample was analyzed by Head-Space Gas Chromatography (HS-GC). The collected data was compared to data collected for certified reference standards at known concentrations.

71387-VC

Compound	CAS	Amount [1]	Limit [2]	RL	Status
Propane	74-98-6	ND	1,000 ppm	100	PASS
Isobutane	75-28-5	ND	1,000 ppm	100	PASS
Butane	106-97-8	ND	1,000 ppm	100	PASS
Methanol	67-56-1	ND	3,000 ppm	100	PASS
Pentane	109-66-0	ND	5,000 ppm	100	PASS
Ethanol	64-17-5	ND	5,000 ppm	100	*
Acetone	67-64-1	ND	5,000 ppm	100	PASS
Isopropanol	67-63-0	ND	5,000 ppm	100	PASS
Acetonitrile	75-05-8	ND	410 ppm	100	PASS
Hexane	110-54-3	ND	290 ppm	100	PASS
Heptane	142-82-5	ND	5,000 ppm	100	PASS

1) ND = Not detected at a level greater than the Reporting Limit (RL).
2) In ppm, based on USP recommended limits for residual solvents, adopted by the Massachusetts Department of Public Health for cannabis concentrates and extracts on 3/31/16. Butane/Propane limits are based on limits established for state of Colorado.
(*) For ethanol, as many formulations contain flavorings based on ethanol extracts of natural products, no status has been assigned.

END OF REPORT

420 Fortune Blvd • Milford, MA 01757 • 617-221-3356
www.ProVerdeLabs.com
